# Sex-Based Performance Disparities in Machine Learning Algorithms for Cardiac Disease Prediction: Exploratory Study

**DOI:** 10.2196/46936

**Published:** 2024-08-26

**Authors:** Isabel Straw, Geraint Rees, Parashkev Nachev

**Affiliations:** 1 University College London London United Kingdom

**Keywords:** artificial intelligence, machine learning, cardiology, health care, health equity, medicine, cardiac, quantitative evaluation, inequality, cardiac disease, performance, sex, management, heart failure

## Abstract

**Background:**

The presence of bias in artificial intelligence has garnered increased attention, with inequities in algorithmic performance being exposed across the fields of criminal justice, education, and welfare services. In health care, the inequitable performance of algorithms across demographic groups may widen health inequalities.

**Objective:**

Here, we identify and characterize bias in cardiology algorithms, looking specifically at algorithms used in the management of heart failure.

**Methods:**

Stage 1 involved a literature search of PubMed and Web of Science for key terms relating to cardiac machine learning (ML) algorithms. Papers that built ML models to predict cardiac disease were evaluated for their focus on demographic bias in model performance, and open-source data sets were retained for our investigation. Two open-source data sets were identified: (1) the University of California Irvine Heart Failure data set and (2) the University of California Irvine Coronary Artery Disease data set. We reproduced existing algorithms that have been reported for these data sets, tested them for sex biases in algorithm performance, and assessed a range of remediation techniques for their efficacy in reducing inequities. Particular attention was paid to the false negative rate (FNR), due to the clinical significance of underdiagnosis and missed opportunities for treatment.

**Results:**

In stage 1, our literature search returned 127 papers, with 60 meeting the criteria for a full review and only 3 papers highlighting sex differences in algorithm performance. In the papers that reported sex, there was a consistent underrepresentation of female patients in the data sets. No papers investigated racial or ethnic differences. In stage 2, we reproduced algorithms reported in the literature, achieving mean accuracies of 84.24% (SD 3.51%) for data set 1 and 85.72% (SD 1.75%) for data set 2 (random forest models). For data set 1, the FNR was significantly higher for female patients in 13 out of 16 experiments, meeting the threshold of statistical significance (–17.81% to –3.37%; *P*<.05). A smaller disparity in the false positive rate was significant for male patients in 13 out of 16 experiments (–0.48% to +9.77%; *P*<.05). We observed an overprediction of disease for male patients (higher false positive rate) and an underprediction of disease for female patients (higher FNR). Sex differences in feature importance suggest that feature selection needs to be demographically tailored.

**Conclusions:**

Our research exposes a significant gap in cardiac ML research, highlighting that the underperformance of algorithms for female patients has been overlooked in the published literature. Our study quantifies sex disparities in algorithmic performance and explores several sources of bias. We found an underrepresentation of female patients in the data sets used to train algorithms, identified sex biases in model error rates, and demonstrated that a series of remediation techniques were unable to address the inequities present.

## Introduction

### Background

Artificial intelligence (AI) has been proposed as an effective solution to many health care challenges and depends on the construction of machine learning (ML) algorithms from health care data. Recent research has drawn attention to the possibility that algorithms may exhibit bias when applied to different demographic groups [1-6]. Such biases may widen health inequalities and negatively impact marginalized patients, such as female patients, minoritized racial and ethnic groups, and other neglected subpopulations [1-7].

Over the past 5 years, an increasing number of studies have quantified disparities in algorithmic performance for underserved populations [2-7]. Daneshjou and colleagues [2] demonstrated that state-of-the-art dermatology algorithms tend to perform worse on darker skin tones; Seyyed-Kalantari and colleagues [3] exposed biases in radiology algorithms; and Thompson and colleagues [4] reported increased false negative errors when classifying opioid misuse disorder for Black patients compared to White patients. Beyond specific diagnoses, researchers have demonstrated that infrastructural AI systems used in hospital settings can be subject to referral bias, demonstrated by Obermeyer and colleagues [5] who highlighted a hospital treatment allocation algorithm that overlooked the health needs of Black patients. Yet despite the increasing number of papers describing this issue, most of the current uses of biomedical AI technologies do not account for the problem of bias [5-8]. Here, we evaluate algorithmic inequity in ML algorithms used for predicting cardiac disease, focusing on heart failure (HF).

### ML for HF

HF is a clinical syndrome in which the heart is unable to maintain a cardiac output adequate to meet the metabolic demands of the body [9]. Traditionally, algorithmic tools capable of identifying at-risk patients have played a key role in informing decisions on HF management and end-of-life care [10-12]. In recent years, ML algorithms that leverage biochemical data have been proposed as a superior alternative to traditional statistical models for identifying at-risk patients with HF [13]. A range of ML techniques outperforms traditional risk scores in forecasting HF-related events [13]. Yet given that existing medical research has described sex differences in both the presentation and management of HF, algorithms trained on existing data may perform differently for male versus female patients [14,15].

### Sex Differences in HF

HF presents differently in female patients compared with male patients [14]. Female patients experience a wider range of symptoms, including higher fluid overload and lower health-related quality of life [14,15]. Moreover, female patients who present with HF are on average older, sustain a higher ejection fraction (EF) throughout later stages of the disease, and have a lower incidence of previous ischemic heart disease [15]. Furthermore, the biochemical tests used to detect cardiac disease have been demonstrated to perform less well for female patients [16]. Troponin is 1 key biomarker used to predict disease, which has been demonstrated to be less sensitive in female patients [16]. Standard troponin criteria fail to detect 1 out of 5 acute myocardial infarcts occurring in female patients [16]. Historically, the neglect of sex differences in cardiac pathophysiology has disadvantaged female patients, and if not considered during ML development, these inequities may manifest in the novel algorithms being integrated into cardiac care [14-19].

In our research, we scope the published literature reporting algorithms that predict HF and investigate whether existing papers give attention to bias in ML algorithms. Furthermore, we examine the data sets of existing models for demographic representation, evaluate demographic inequities in algorithmic performance, and assess the efficacy of a series of bias-mitigation techniques.

## Methods

### Study Design

**Figure 1:**
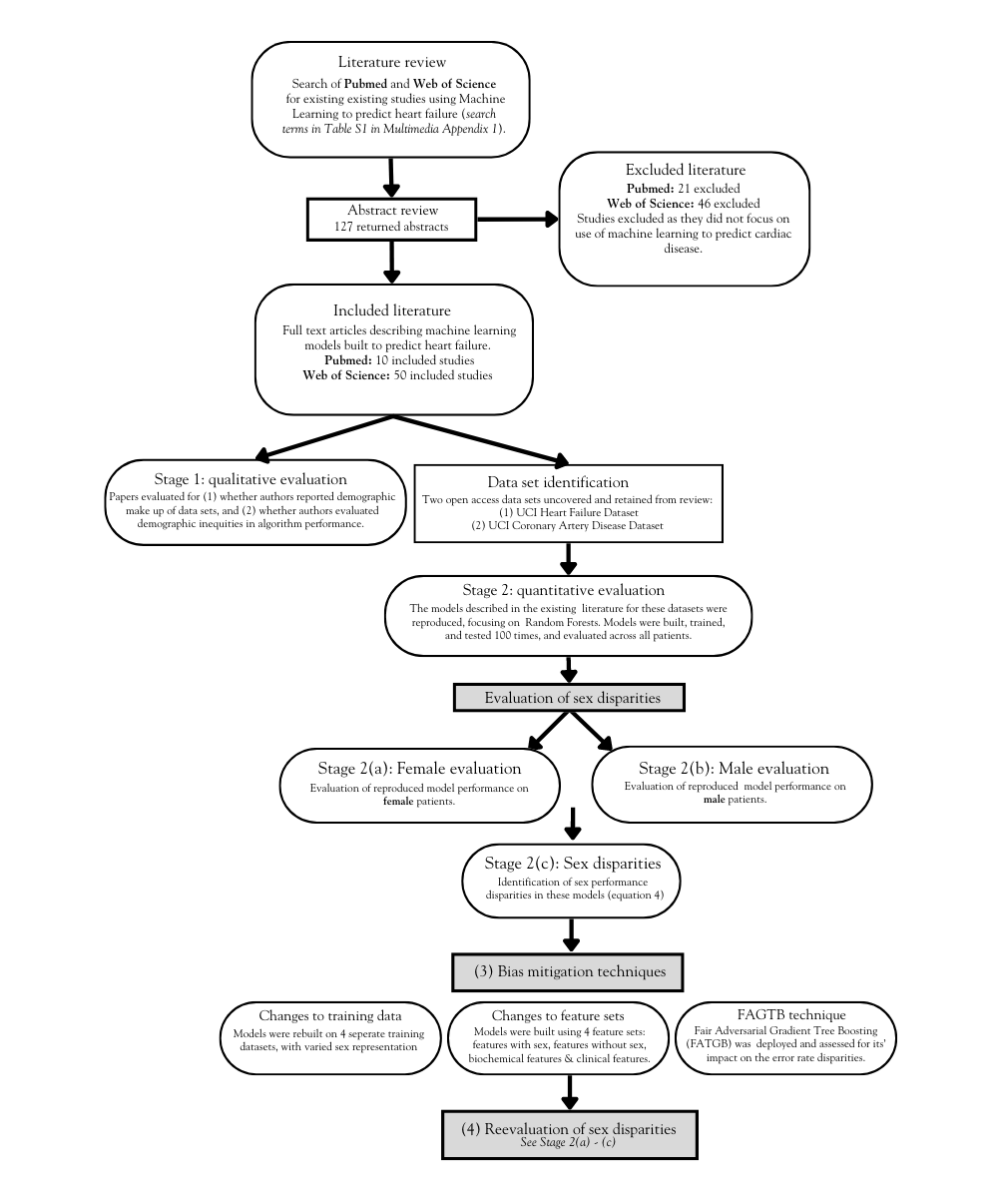
A flowchart detailing the steps of our methodology, including (1) the initial literature search and qualitative evaluation of identified studies and (2) the identification of data sets and interrogation of algorithms for demographic bias. FAGTB: Fair Adversarial Gradient Tree Boosting; HF: heart failure; ML: machine learning; UCI: University of California Irvine.

Our analysis consists of two stages: (1) a literature review of papers describing ML models used to predict HF and (2) a quantitative analysis of identified models, evaluating inequities in algorithm performance. The flowchart in Figure 1 provides an overview of our approach.

### Stage 1 Literature Review: Qualitative Evaluation of Published Papers

**Figure 2:**
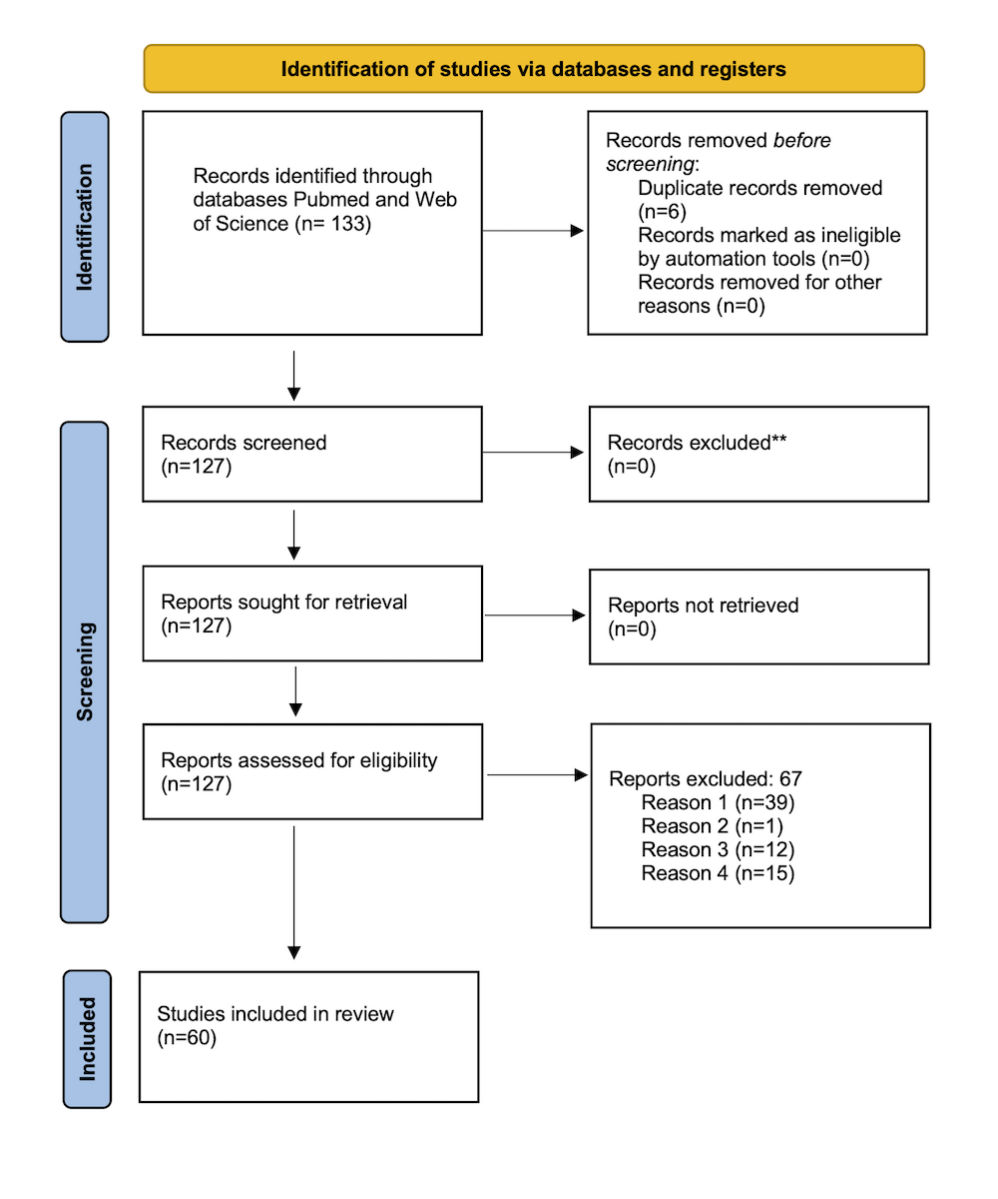
PRISMA 2020 flow diagram for new systematic reviews which included searches of databases and registers only (PRISMA templated obtained from PRISMA at https://prisma-statement.org/prismastatement/flowdiagram.aspx) **Reasons for exclusion: Reason 1: The study did not focus on biochemical data or laboratory tests, instead utilizing different modalities (eg, visual data from radiological scans); Reason 2: The study did not use machine learning techniques (eg, it used traditional statistical methods); Reason 3: The study did not describe empirical research, involving the development of ML models for prediction of cardiac disease (eg, instead the paper was a review or commentary); Reason 4: The retrieved study was not a full paper, instead it was a conference or meeting abstract.

We searched PubMed and Web of Science between April 1, 2022, and May 22, 2022, to identify ML algorithms used to predict cardiac disease adhering to PRISMA (Preferred Reporting Items for Systematic Reviews and Meta-Analyses) guidelines for systematic reviews (Figure 2 [20] and Tables S1 and S2 in Multimedia Appendix 1 [21,22]). All abstracts were reviewed, and papers were included for full-text review if they met the following criteria: (1) the target diagnosis was HF, (2) the model used biochemical markers to predict disease, and (3) the computational methods involved an ML approach (including supervised, unsupervised, and deep learning).

Of the retained papers, full texts were then reviewed to evaluate whether authors (1) reported the demographic make-up of data sets and (2) evaluated demographic inequities in algorithm performance, meaning that the authors specifically examined differences in algorithmic performance by demographic groups defined by protected characteristics [17].

Throughout the literature review, any identified open-source data sets were maintained for use in stage 2.

### Stage 2: Quantitative Evaluation of Model Performance

Two open-source data sets were uncovered in our literature review: (1) data set 1: University of California Irvine for Heart Failure Prediction [21] and (2) data set 2: University of California Irvine Cleveland Heart Disease data set for identifying coronary artery disease (CAD) [22]. Descriptive statistics were performed on both data sets, evaluating the mean and variance of the data set variables for sexes separately, affected by disease or death (Table 1 and Tables S3-S5 in Multimedia Appendix 1).

**Table 1:** Descriptive statistics of the variables in data set 1 (heart failure; N=299), stratified by target (death) and sex^a^.

Variables	Sex and death (target variable)^b^			
	Female (sex=0; n=105)		Male (sex=1; n=194)	
	Survived (HF^c^ death=0)	Death (HF death=1)	Survived (HF death=0)	Death (HF death=1)
Total count, n (%)	71 (67.62)	34 (32.38)	132 (68.04)	62 (32.96)
Age (years), mean (SD)	58.6 (10.6)	62.2 (12.3)	58.8 (10.7)	66.9 (13.5)
Anemia (Boolean), mean (SD)	0.5 (0.5)	0.6 (0.5)	0.4 (0.5)	0.4 (0.5)
Creatinine phosphokinase (mcg/L), mean (SD)	462.0 (517.7)	507.7 (779.7)	582.8 (853.2)	759.3 (1532.3)
Diabetes mellitus (Boolean), mean (SD)	0.5 (0.5)	0.6 (0.5)	0.4 (0.5)	0.3 (0.5)
Ejection fraction (percentage), mean (SD)	41.9 (11.6)	37.5 (14.6)	39.4 (10.4)	31.2 (10.7)
High blood pressure (Boolean), mean (SD)	0.4 (0.5)	0.5 (0.5)	0.3 (0.5)	0.4 (0.5)
Platelets (kiloplatelets/mL), mean (SD)	289,757.6 (98,655.9)	259,512.7 (107,588.6)	254,232.4 (94,985.6)	254,663.7 (94,060.8)
Serum creatinine (mg/dL), mean (SD)	1.1 (0.6)	1.9 (1.6)	1.2 (0.7)	1.8 (1.4)
Serum sodium (mEq/L), mean (SD)	137.4 (3.6)	135.5 (6.7)	137.1 (4.2)	135.3 (3.8)
Smoking (Boolean), mean (SD)	0.0 (0.1)	0.1 (0.3)	0.5 (0.5)	0.4 (0.5)

^a^Full details of data set variables are available in Tanvir et al [21].

^b^For the death variable, a value of 1 indicates mortality.

^c^HF: heart failure.

Using these data sets, we rebuilt the ML algorithms described in the published literature and performed an additional analysis exploring inequities in algorithmic performance for demographic subgroups. As the only protected characteristic reported was sex, we focus on sex disparities in performance. Despite our initial aim to focus on HF, we retained an uncovered CAD data set to investigate whether trends identified for HF generalized to patients with CAD [22]. Tables S3 and S4 in Multimedia Appendix 1 provide details on data set 1 and data set 2, respectively.

### Model Reproduction

We rebuilt the models described in the existing literature for these data sets, focusing on random forest (RF) algorithms, which have been widely reported to be the most effective models [23]. For both data sets, data was split into test or training subsets (0.7:0.3), RF models were built using SciKit Learn, and RF parameters were tuned using GridSearch CV (SciKit Learn). We adopted a bootstrapping approach to quantify uncertainty, such that models were built, trained, and tested 100 times, from which average results were derived with SD.

### Statistical Analysis

Across the 100 runs, sex differences in each algorithm evaluation metric (equations 1-10) were calculated and averaged, with accompanying statistical tests performed to evaluate for statistical significance of any identified sex disparities. Our method for examining differences in algorithmic error rates builds on the foundational work from Buolamwini and Gebru [24], who demonstrated that a range of ML algorithms for facial recognition performed poorly on darker-skinned female patients. To evaluate for statistical significance, independent 2-tailed *t* tests were performed where the data was normally distributed, and Mann-Whitney *U* tests were performed where the data was not normally distributed. Kolmogorov-Smirnov tests were used to assess for normality [25].

### Variations in Model Development

#### Overview

We then introduced a variety of changes to the model development, to evaluate the impact on the identified sex disparities in performance.

#### Changes to Model Training Data

In total, 1 widely proposed bias mitigation technique includes preprocessing the training data of a model to account for demographic representation, with previous research highlighting the benefit of training on demographically balanced or demographically stratified data sets [26]. We therefore created a range of data sets with varied sex representation and assessed for the impact on algorithm performance disparities. To form the sex-balanced data set, we used the oversampling function of *SMOTE()*, which has been proposed as an effective method for improving the representation of underserved populations in ML data sets [27]. The *SMOTE* package generates new minority data points based on existing minority samples through linear interpolation [26,27]. Models were rebuilt as per the *Model Reproduction* section, using 4 different training data sets (sex-imbalanced, sex-balanced, and sex-specific; Tables S6 and S7 in Multimedia Appendix 1): (1) original sex-imbalanced training data, (2) sex-balanced training data, (3) female-only training data, and (4) male-only training data experiments.

#### Changes to Feature Selection

To understand why models make certain decisions, researchers in the domain of “explainable AI” have demonstrated how feature evaluation may provide important information regarding model performance for different subpopulations [26,28]. To do this, Shapley values have been widely accepted as a unified measure of feature importance since their proposal in 2017 [29].

In our experiments, we first perform an exploratory analysis, comparing feature importance for models trained on the male versus female data sets. Second, we create 4 feature subsets from the original data sets, to evaluate the impact of changing the feature selection on performance disparities. As described in the introduction, existing clinical research has described demographic differences in the biochemical and clinical markers of HF disease (eg, sex differences in EF and troponin levels) [16]. Thus, we delineate 4 different feature subsets that vary in this information, to examine whether certain feature subsets perform better for different demographic groups. These four feature subsets are described in detail in Tables S8 and S9 in Multimedia Appendix 1 and include (1) features with sex, (2) features without sex, (3) biochemical features, and (4) clinical features.

Our final series of experiments are therefore performed across the four training data sets (sex-imbalanced, sex-balanced, and sex-specific), and the four feature sets giving 16 total experiments: (1) original sex-imbalanced training data experiments (across four feature subsets), (2) sex-balanced training data experiments (across four feature subsets), (3) female training data experiments (across four feature subsets), and (4) male training data experiments (across four feature subsets)

### Model Evaluation and Identification of Performance Disparities

Models are evaluated using global evaluation metrics (eg, accuracy) and specific error rates (eg, false negative rate [FNR]; equations 1-10). The difference between male and female scores is calculated to give a model’s “sex performance disparity” (equation 10). To evaluate for statistical significance, Kolmogorov-Smirnov Tests were used to assess for the normality of the data, following which independent 2-tailed *t* tests were performed where the data were normally distributed, and Mann-Whitney *U* tests were performed where the data were not normally distributed.

Our choice of evaluation metrics is guided by the clinical consequence of each of these scores.

The existing research on algorithmic bias has highlighted the importance of examining error rates, particularly in medicine where a false negative clinically translates to missed diagnoses or opportunities for treatment [3-6,26]. As described by Afrose and colleagues [26], focusing on global metrics of performance such as area under the receiver operating characteristic curve scores can neglect subtler disparities arising from differences in error rates affecting subgroups. When selecting a bias assessment metric, previous studies have chosen to focus on FNR and false positive rate (FPR), due to the clinical implications of these errors [4,30,31]. Equations 5-8 places the error rates in their clinical context, demonstrating that the FNR represents missed diagnoses and potentially missed treatment. For the error rates, we use the threshold of 0.5, as we are investigating performance inequities in the existing reported models that used these default settings.

Error rate definitions are as follows:

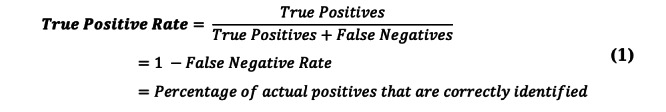

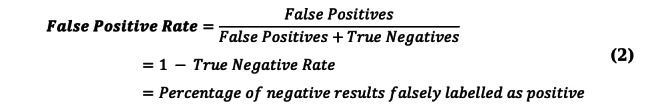

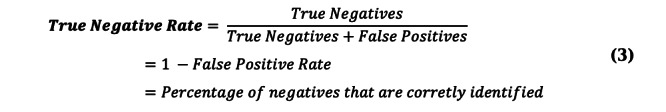

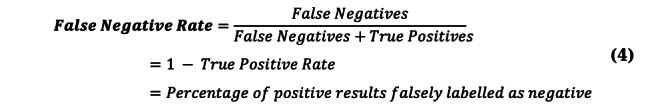









Clinical implications of error rates are as follows:


*True Positive Rate = Correct diagnosis that patient as disease*
**(5)**

*False Positive Rate = Misdiagnosis of disease when patient is healthy*
**(6)**

*True Negative Rate = Correct diagnosis that patient is healthy*
**(7)**

*False Negative Rate = Misdiagnosis that patient is healthy when patient has disease*
**(8)**

The accuracy evaluation metric is calculated as follows:

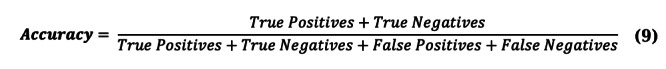



Sex performance disparity is calculated as follows:


*Sex performance disparity = Score for male patients (mean) – Score for female patients (mean)*
**(10)**

### Fairness Techniques: Fair Adversarial Gradient Tree Boosting

We implemented a recent fairness technique to evaluate whether these approaches applied to bias in HF algorithms. The Fair Adversarial Gradient Tree Boosting (FAGTB) is a recent technique proposed by Grari et al [8] for mitigating bias in decision tree classifiers and the authors demonstrate the success of their technique on 4 data sets. The authors focus on 2 definitions of fairness: demographic parity and equalized odds [8]. The equalized odds metric focuses on model FPR and FNR, and hence we highlight this for our paper. A summary of these fairness metrics is provided in Section S1 in Multimedia Appendix 2 for further interest.

The definition of equalized odds is as follows:

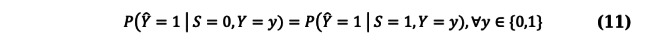



To assess for the equalized odds the authors measure the disparate mistreatment, which computes the absolute difference between FPR and the FNR for both demographics.

The disparate FPR is calculated as follows:

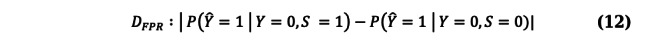



The disparate FNR is calculated as follows:

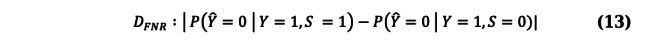



We compare the performance of the FAGTB algorithm to a standard Gradient Tree Algorithm. As per the original FAGTB paper, we repeat 10 experiments randomly sampling 2 subsets (0.8:0.2) and report evaluation metrics for the test set.

### Ethical Considerations

Ethical approval was not required for this study as all data used were sourced from publicly available open-source data sets [21,22] under a CC-BY 4.0 license. No direct patient contact or sensitive personal data was involved, ensuring compliance with research standards.

## Results

### Literature Review Search Results

Our search returned 127 papers, of which 60 met the criteria for full review and 3 highlighted sex differences in model performance. In the papers that reported sex, there was a consistent underrepresentation of female patients. No papers investigated racial or ethnic differences. Further, 1 paper focused specifically on female patients with HF, in which Tison et al [32] highlighted that HF was more common in people who were older, White, with a higher mean number of pregnancies, a higher BMI, and were less likely to have Medicare.

### Descriptive Statistics and Feature Importance

#### Data Set 1 (HF)

The mean descriptive statistics for each feature present in the HF data set are provided in Table 1, which demonstrates subtle sex differences in the presentation of the disease. For HF deaths, male patients tend to be older than their female counterparts, with a higher creatinine phosphokinase, lower likelihood of diabetes, lower EF, and lower blood pressure.

**Figure 3:**
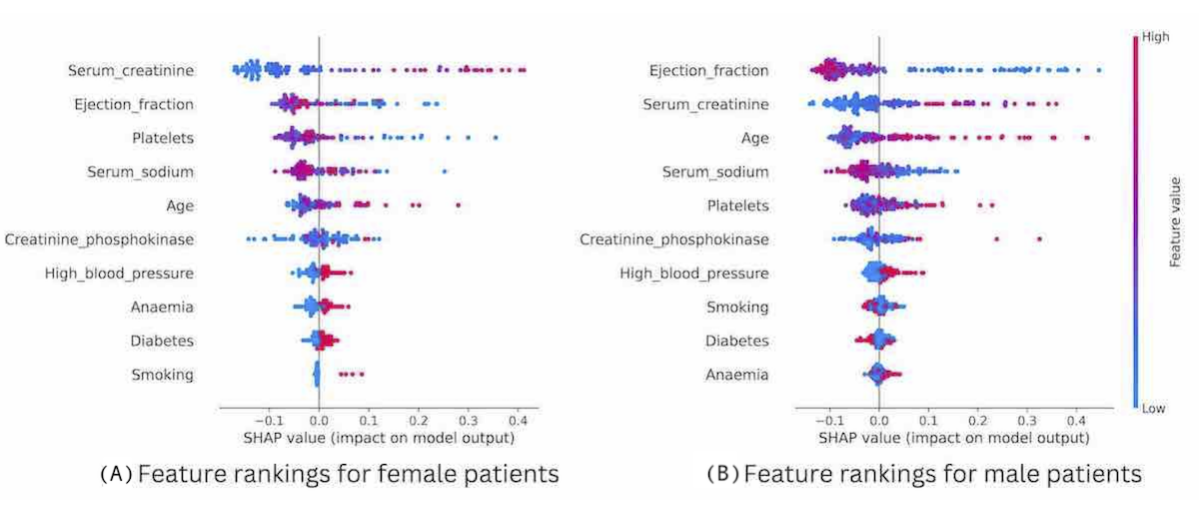
Comparison of feature rankings for male and female patients, ordered by SHA*P* values. SHAP: Shapley additive explanations.

Our exploratory analysis identified further sex differences on examining feature importance. Figure 3 compares the rankings of feature importance for ML models built to predict HF built from the female data set compared to the male data set. These differences are important as existing ML algorithms built on mixed-sex cohorts suggest that EF can be used alone for modeling, an approach that may disadvantage female patients [23].

#### Data Set 2 (CAD)

Table S5 in Multimedia Appendix 1 provides details of the CAD data set and demonstrates that female patients with CAD have higher resting blood pressure and higher cholesterol compared to male patients. The categorical variable “resting electrocardiogram” is also higher for female patients, due to a higher incidence of left ventricular hypertrophy.

### Model Results and Performance Disparities

We replicated the algorithms described in the existing literature, reproducing the same previously reported mean predictive accuracies of 84.24% (3.51 SD) for data set 1 and 85.72% (1.75 SD) for data set 2 [23]. In Tables 2 and 3, we present the disparity in performance for the sexes, where a positive value indicates a higher value for male patients (see equation 10).

**Figure 4:**
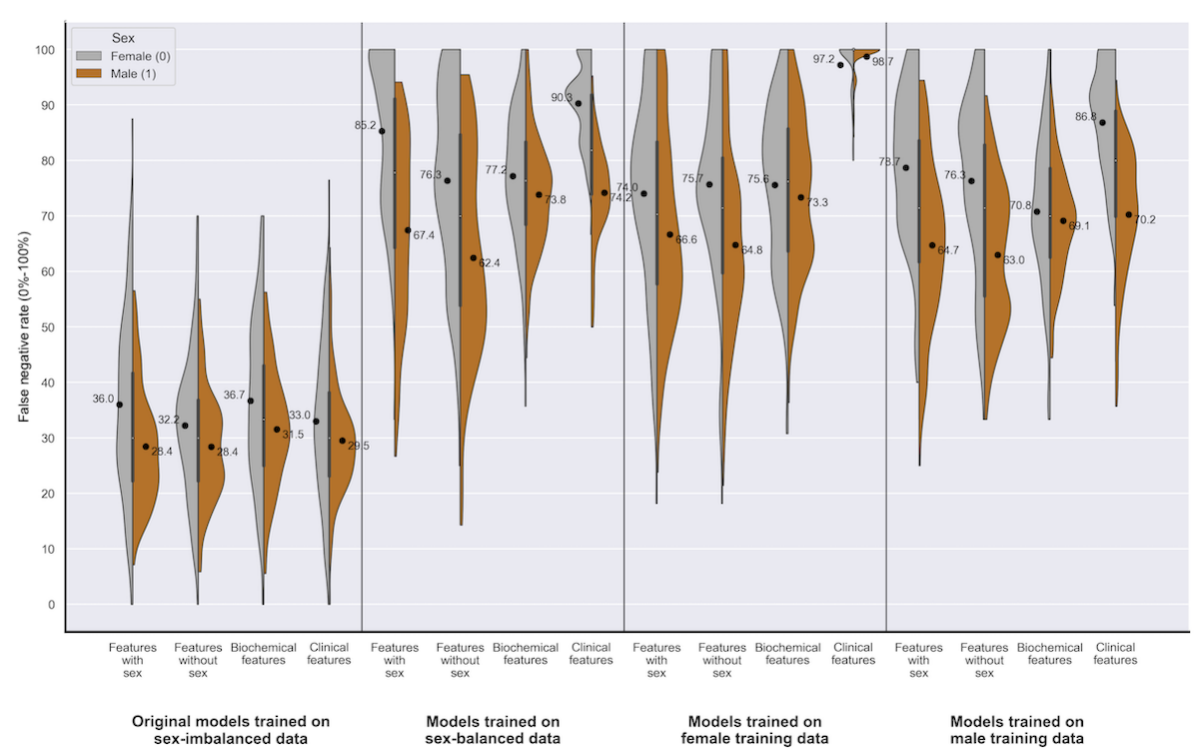
Data set 1 (heart failure): a series of violin plots showing the sex-stratified performance (false negative rate [0%-100%]) of the random forests trained across the 4 feature sets, on the different variations in training data. The plots show male (orange) and female (gray) FNR alongside each other, in groups of 4 (divided by a line) according to the training data used (sex-imbalanced, sex-balanced, female, and male). The feature set used is indicated within each training data group (features with sex, features without sex, biochemical features, and clinical features). See Multimedia Appendixes.

For data set 1, Table 2 demonstrates that in 13 out of 16 experiments, the FNR is higher for female patients, meeting the threshold of statistical significance (mean difference of –17.81% to –3.37%; *P*<.05). Figure 4 represents this disparity in performance graphically, providing the point estimates of FNR for the sexes separately and highlighting that the disparity in FNR persisted across the variations in training data and selected features.

**Table 2:** Sex performance disparities for models built from data set 1 (heart failure disease)–sex performance disparities are calculated as the performance for male patients minus the performance for female patients (see equation 10). Thus, a positive value indicates a higher score for male patients and a negative value indicates a higher score for female patients. All disparities are presented alongside results of significance testing, where significant differences between the sexes are highlighted with a footnote (*P*<.05).

Disparity in model performance (score for male patients – score for female patients)		Feature subset used in model training							
		Features with sex	*P* value	Features without sex	*P* value	Biochemical features	*P* value	Clinical features	*P* value
**Sex-imbalanced training data**									
	Accuracy disparity (%)	1.63	.03^a^	–0.72	.30	0.10	.88	–0.50	.49
	ROC_AUC^b^ disparity (%)	3.14	<.01^a^	0.43	.61	1.51	.09	0.47	.60
	FNR^c^ disparity (%)	–7.53	<.01^a^	–3.84	.02^a^	–5.15	.01^a^	–3.49	.049^a^
	FPR^d^ disparity (%)	1.26	.07	2.97	<.01^a^	2.11	<.01^a^	2.56	<.01^a^
**Sex-balanced training data**									
	Accuracy disparity (%)	–4.78	<.01^a^	–7.25	<.01^a^	–9.42	<.01^a^	–3.63	<.01^a^
	ROC_AUC disparity (%)	7.0	<.01^a^	4.27	<.01^a^	0.15	.83	8.32	<.01^a^
	FNR disparity (%)	–17.81	<.01^a^	–13.91	<.01^a^	–3.37	.04^a^	–16.09	<.01^a^
	FPR disparity (%)	3.90	<.01^a^	5.37	<.01^a^	3.07	<.001^a^	–0.54	.24
**Female training data**									
	Accuracy disparity (%)	–10.95	<.01^a^	–9.75	<.01^a^	–12.32	<.01^a^	–9.64	<.01^a^
	ROC_AUC disparity (%)	0.60	.57	0.57	.23	–2.92	<.01^a^	–0.53	.07
	FNR disparity (%)	–7.42	<.01^a^	–10.91	<.01^a^	–2.24	.27	1.55	.01^a^
	FPR disparity (%)	8.61	<.01^a^	9.77	<.01^a^	8.08	<.01^a^	–0.48	.04^a^
**Male training data**									
	Accuracy disparity (%)	–5.46	<.01^a^	–5.73	<.01^a^	–8.73	<.01^a^	–2.46	<.01^a^
	ROC_AUC disparity (%)	4.98	<.01^a^	4.54	<.01^a^	–1.59	.049^a^	8.32	<.01^a^
	FNR disparity (%)	–13.96	<.01^a^	–13.32	<.01^a^	–1.68	.33	–16.58	<.01^a^
	FPR disparity (%)	4.00	<.01^a^	4.24	<.01^a^	4.86	<.01^a^	–0.06	.35

^a^Indicates a statistically significant difference (*P*<.05) between the model’s performance on male versus female patients.

^a^ROC_AUC: area under the receiver operating characteristic curve.

^b^FNR: false negative rate.

^c^FPR: false positive rate.

**Table 3:** Sex performance disparities for models built from data set 2 (coronary artery disease)—sex performance disparities are calculated as the performance for male patients minus the performance for female patients (see equation 10). Thus, a positive value indicates a higher score for male patients, and a negative value indicates a higher score for female patients. All disparities are presented alongside results of significance testing, where significant differences between the sexes are highlighted with a footnote (*P*<.05).

Disparity in model performance (score for male patients – score for female patients)		Feature subset used in model training							
		Features with sex	*P* value	Features without sex	*P* value	Biochemical features	*P* value	Clinical features	*P* value
**Sex-imbalanced training data**									
	Accuracy disparity (%)	0.32	.50	0.64	.17	0.13	.80	0.25	.61
	ROC_AUC^b^ disparity (%)	3.86	<.01^a^	4.24	<.01^a^	3.05	<.01^a^	3.91	<.01^a^
	FNR^c^ disparity (%)	–11.66	<.01^a^	–12.52	<.01^a^	–10.81	<.01^a^	–12.38	<.01^a^
	FPR^d^ disparity (%)	3.94	<.01^a^	4.04	<.01^a^	4.71	<.01^a^	4.57	<.01^a^
**Sex-balanced training data**									
	Accuracy disparity (%)	–4.01	<.01^a^	–5.12	<.01^a^	–7.32	<.01^a^	–2.86	<.01^a^
	ROC_AUC disparity (%)	–3.89	.01^a^	–4.91	.01^a^	–7.18	<.001^a^	–2.75	<.01^a^
	FNR disparity (%)	7.69	<.01^a^	10.54	<.01^a^	15.59	<.01^a^	6.61	<.01^a^
	FPR disparity (%)	0.10	.87	–0.72	.19	–1.23	.29	–1.11	.06
**Female training data**									
	Accuracy disparity (%)	–9.25	<.01^a^	–11.34	<.01^a^	–11.49	<.01^a^	–8.69	<.01^a^
	ROC_AUC disparity (%)	–8.97	<.01^a^	–10.95	<.01^a^	–11.10	<.01^a^	–8.45	<.01^a^
	FNR disparity (%)	18.98	<.01^a^	22.60	<.01^a^	27.23	<.01^a^	17.86	<.01^a^
	FPR disparity (%)	–1.04	.07	–0.70	.20	–5.02	<.01^a^	–0.96	.09
**Male training data**									
	Accuracy disparity (%)	6.38	<.01^a^	5.66	<.01^a^	–1.66	.02^a^	6.10	<.01^a^
	ROC_AUC disparity (%)	6.30	<.01^a^	5.57	<.01^a^	1.52	.07	5.86	.01^a^
	FNR disparity (%)	–10.12	<.01^a^	–10.10	<.001^a^	1.67	.17	–12.64	<.01^a^
	FPR disparity (%)	–2.48	<.01^a^	–1.04	.07	1.38	.24	0.92	.15

^a^Indicates a statistically significant difference (*P*<.05) between the model’s performance on male versus female patients. To determine statistical significance, the Kolmogorov-Smirnov tests were first run on the sex-stratified results to determine the distribution of data (normal or not). Independent 2-tailed *t* tests were used where data were normally distributed, and Mann-Whitney *U* tests were used when data were not normally distributed.

^b^ROC_AUC: area under the receiver operating characteristic curve.

^c^FNR: false negative rate.

^d^FPR: false positive rate.

**Figure 5:**
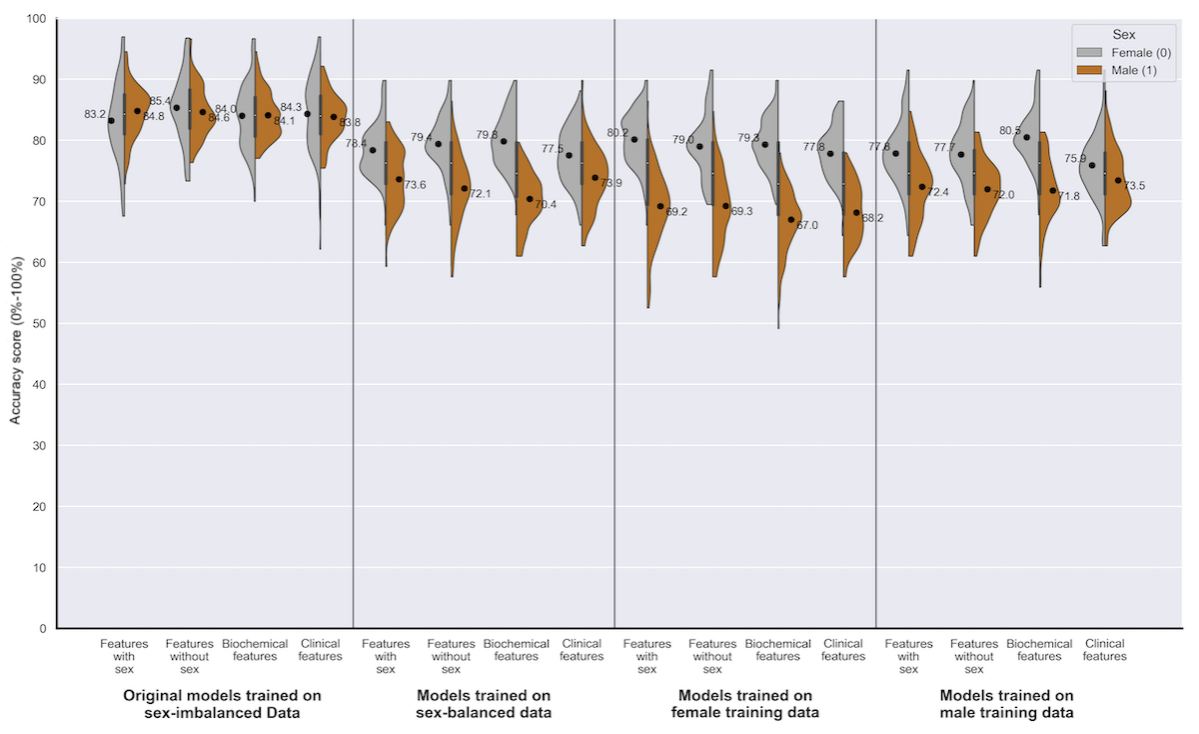
Data set 1 (heart failure): a series of violin plots showing the sex-stratified performance (accuracy [0%-100%]) of the random forests trained across the 4 feature sets, on the different variations in training data. The plots show male (orange) and female (gray) accuracy alongside each other, in groups of 4 (divided by a line) according to the training data used (sex-imbalanced, sex-balanced, female, and male). The feature set used is indicated within each training data group (features with sex, features without sex, biochemical features, and clinical features). See Multimedia Appendixes.

A smaller disparity in the FPR was statistically significant for male patients in 13 out of 16 experiments (–0.48% to +9.77%; *P*<.05). The sex performance disparities in accuracy and area under the receiver operating characteristic curve varied depending on the underlying shifts in the error rates for each sex (Table 2 and Figure 5). On examining the individual error rates, we see consistencies in the sex disparities across feature sets, most notably an overprediction of disease for male patients (higher FPR) and an underprediction of disease for female patients (higher FNR: Table 2).

**Figure 6:**
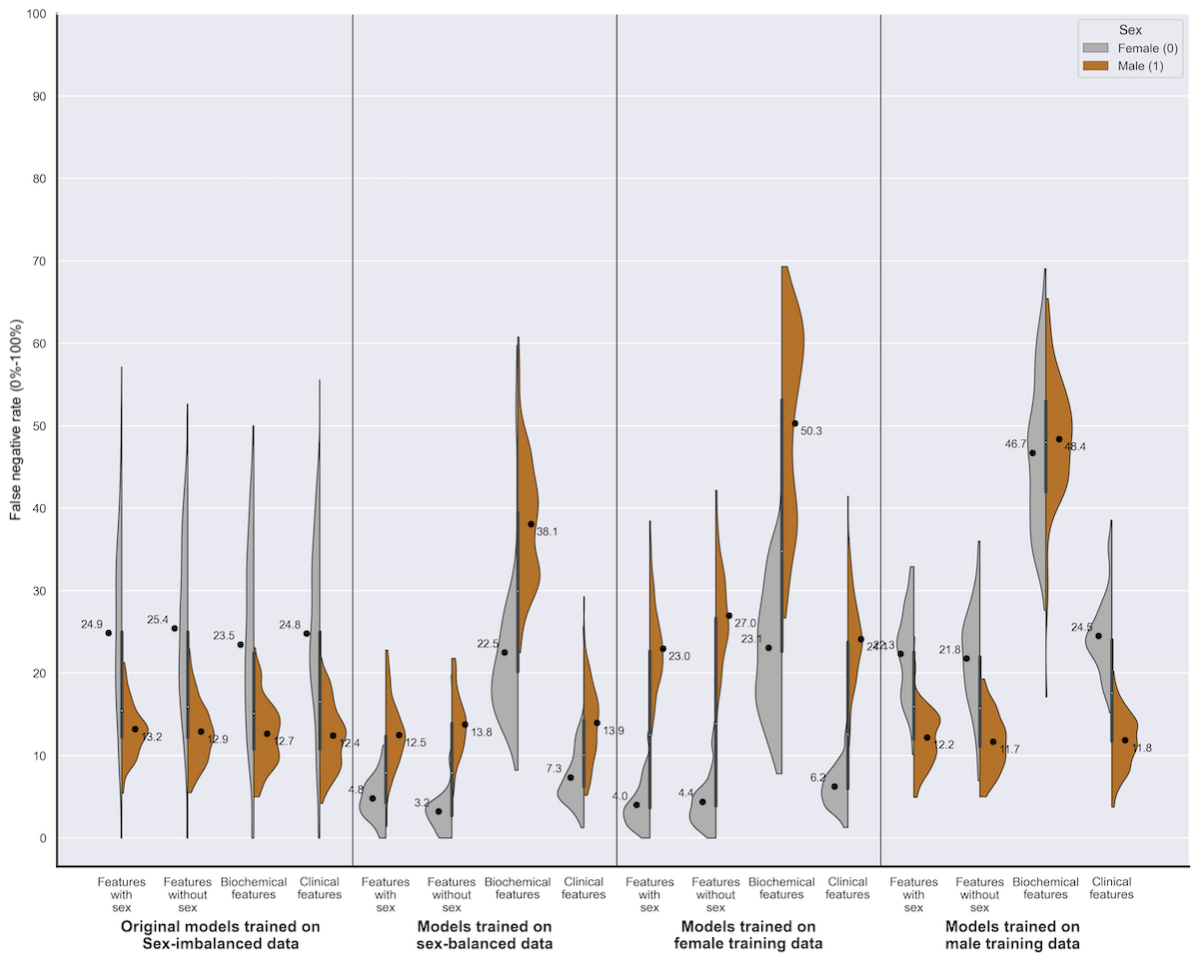
Data set 2 (coronary artery disease): a series of violin plots showing the sex-stratified performance (false negative rate [0%-100%]) of the random forests trained across the 4 feature sets, on the different variations in training data. The plots show male (orange) and female (gray) FNR alongside each other, in groups of 4 (divided by a line) according to the training data used (sex-imbalanced, sex-balanced, female, and male). The feature set used is indicated within each training data group (features with sex, features without sex, biochemical features, and clinical features). See Multimedia Appendixes. FNR: false negative rate.

**Figure 7:**
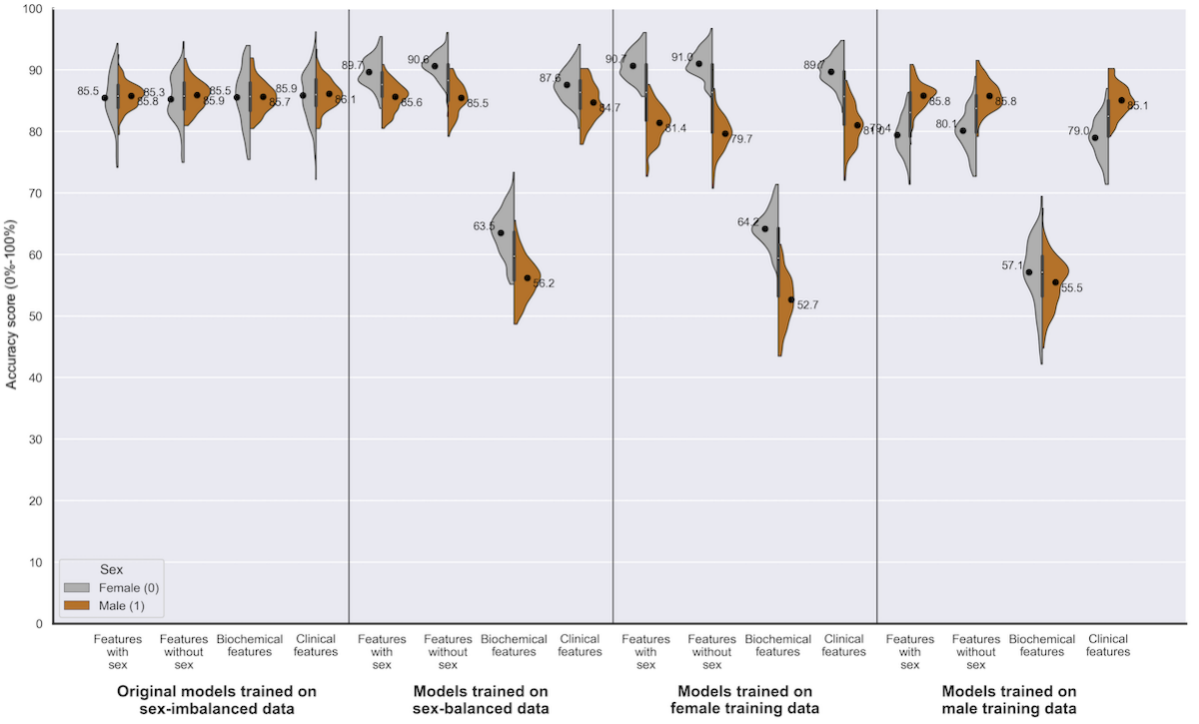
Data set 2 (coronary artery disease): a series of violin plots showing the sex-stratified performance (accuracy [0%-100%]) of the random forests trained across the 4 feature sets, on the variations in training data. The plots show male (orange) and female (gray) accuracy alongside each other, in groups of 4 (divided by a line) according to the training data used (sex-imbalanced, sex-balanced, female, and male). The feature set used is indicated within each training data group (features with sex, features without sex, biochemical features, and clinical features).

Our findings for data set 2 were similar to those for data set 1, such that models built on the original sex-imbalanced data set demonstrated a higher FNR for female patients (mean difference of –10.81% to –12.52%; *P*<.05; Table 3) and a higher FPR for male patients (3.94% to 4.71%; *P*<.05; Table 3). Figure 6 visualizes the disparity graphically, and demonstrates that, unlike data set 1, the disparity in error rates reversed when training on sex-balanced data and female-only data (Figure 6). Figure 7 illustrates the disparity in accuracy between the sexes, where we see that the direction of the disparity varies depending on the training data and feature set (Figure 7).

### Variations in Training Data

#### Sex-Balanced Training Data

Training on sex-balanced data led to a fall in mean accuracy for all patients in data set 1 (76%, SD 3.46% vs 84.24%, SD 3.51%), with a more substantial drop in mean accuracy for male patients (73.61%, SD 4.84% vs 84.84%, SD 4.16%; Table 4 and Figure 5). The opposite trend was seen in data set 2, with models trained on sex-balanced data outperforming models trained on sex-imbalanced data for all patients (87.65%, SD 1.77% vs 85.72%, SD 1.75%) and for female patients (89.66%, SD 2.44% vs 85.48%, SD 4.12%; Table 4). The models trained on sex-balanced data in data set 2 reduced the FNR for both sexes when using the full feature set (female patients 4.79%, SD 2.58% vs 24.86%, SD 11.35%; male patients 12.48%, SD 4.11% vs 13.19%, SD 3.26%; Table 4 and Figure 6). The differences between the data sets may relate to underlying differences in the 2 cardiac conditions. Further, the failure to improve performance with sex-balanced training data may reflect the issues of mixing data that has conflicting indicators for disease.

**Table 4:** Model results when trained on sex-specific subsets for all patients and male or female patients separately, looking at the “features including sex” subset.

Results	Data set 1 (heart failure)					Data set 2 (coronary artery disease)			
	Sex-imbalanced training data (n=209)	Sex-balanced training data (n=272)	Female training data (n=136)	Male training data (n=136)	Sex-imbalanced training data (n=522)		Sex-balanced training data (n=715)	Female training data (n=358)	Male training data (n=358)
								
All patients, mean accuracy (SD)	84.24 (3.51)	76.0 (3.46)	74.68 (3.53)	75.12 (3.71)	85.72 (1.75)		87.65 (1.77)	86.06 (1.67)	82.63 (1.94)
Female patients, mean accuracy (SD)	83.21 (6.37)	78.39 (19.68)	80.15 (4.43)	77.85 (5.21)	85.48 (4.12)		89.66 (2.44)	90.69 (2.38)	79.44 (3.20)
Male patients, mean accuracy (SD)	84.84 (4.16)	73.61 (4.84)	69.20 (5.96)	72.39 (5.32)	85.80 (2.14)		85.65 (2.23)	81.44 (3.02)	85.82 (2.30).
Female patients, mean FNR^a^ (SD)	35.98 (16.72)	85.25 (14.58)	74.04 (17.68)	78.66 (14.0)	24.86 (11.35)		4.79 (2.58)	4.00 (2.74)	22.32 (5.25)
Male patients, mean FNR (SD)	28.45 (10.41)	67.43 (16.6)	66.62 (17.32)	64.70 (14.9)	13.19 (3.26)		12.48 (4.11)	22.97 (5.20)	12.20 (3.41)

^a^FNR: false negative rate.

#### Sex-Specific Training Data

For data set 1, mean accuracy for all patients when trained on sex-imbalanced data (84.24%, SD 3.51%) falls when training both on female-specific data (74.68%, SD 3.53%) and male-specific training data (75.12%, SD 3.71%), likely related to the smaller training data. For data set 2, mean accuracy for all patients when trained on sex-imbalanced data (85.72%, SD 1.75%) improves when training on female-specific data (86.06%, SD 1.67%) and falls when training on male-specific training data (82.62%, SD 1.94%). The overall improvement seen in the data set 2 models when trained on female data, relates to the increase in accuracy for female patients (90.69%, SD 2.38% vs 85.48%, SD 4.12%) co-occurring with a smaller decrease in accuracy for male patients (81.44%, SD 3.02% vs 85.80%, SD 2.14%; Table 3 and Figure 7).

Unsurprisingly, performance for each sex is lowest when trained on the opposing sex (Table 4, Figures 4-7). In data set 1, same-sex training was preferable to opposite-sex training; however, this did not improve results compared to the models built from sex-imbalanced and sex-balanced training data, likely relating to the smaller sample size (Table 4). In contrast, data set 2 had greater training data available and demonstrated that sex-specific training is beneficial to both sexes above the sex-imbalanced models (Table 4).

#### Variations in Feature Sets

Models built on the biochemical features subset gave the worst performance in terms of accuracy and FNR (Figures 4-7). For data set 2, biochemical features included just cholesterol and fasting blood sugar, and so, the fall in performance may relate to information loss. Additionally, Table S5 in Multimedia Appendix 1 highlights the different biochemical profiles for male and female patients who were sick, with female patients who were sick demonstrating a far higher cholesterol level than their male counterparts (mean values: 279.2 female patients who were sick vs 247.5 male patients who were sick).

#### FAGTB Model

The disparity in false negative rate (DispFNR) was consistently higher than the disparity in false positive rate (Table 5). Compared to the Gradient Boosting Classifier, the FAGTB reduced the DispFNR for both data sets (data set 1: 0.20 vs 0.21; data set 2: 0.19 vs 0.28), however, the DispFNR that disadvantaged female patients persisted. The fall in DispFNR and disparity in false positive rate that occurred with FAGTB was associated with a fall in overall accuracy for both data sets.

**Table 5:** Results of bias mitigation with Fair Adversarial Gradient Tree Boosting (FAGTB).

Results on test set, averaged over 10 experiments			Gradient boosting classifier		FAGTB
**Data set 1 (heart failure): experiments run on sex-imbalanced data with all features (averaged over 10 experiments)**					
	Accuracy	71.3		71.2	
	DispFPR^a^	0.08		0.08	
	DispFNR^b^	0.21		0.20	
**Data set 2 (coronary artery disease): experiments run on sex-imbalanced data with all features (averaged over 10 experiments)**					
	Accuracy	86.3		82.9	
	DispFPR	0.06		0.06	
	DispFNR	0.28		0.19	

^a^DispFPR: disparity in false positive rate.

^b^DispFNR: disparity in false negative rate.

## Discussion

### Principal Findings

Our study sheds light on an important gap in existing cardiac ML research, with significant implications for digital health equity. We find that the majority of published ML studies predicting HF fail to acknowledge the underrepresentation of female patients in their data sets and do not perform stratified model evaluations, thus failing to assess sex disparities in algorithmic performance. Our secondary evaluation of 2 cardiac data sets exposed a neglected sex disparity in model performance, highlighting the importance of integrating these methods into future studies that use ML methods for cardiac modeling. In our approach, we identified several potential sources of algorithmic bias.

First, we detected the underrepresentation of female patients in training data sets that may produce inequalities in model fidelity. Despite introducing oversampling techniques to address this omission, the disparities in performance persisted suggesting that addressing data set representation alone is not a sufficient measure for mitigating bias. Further, our experiments demonstrated that oversampling could reduce overall performance, which may result from the mixing of conflicting data (ie, male vs female feature rankings). In addition, oversampling with synthetic instances solely from the data set at hand does not provide the machine with more information, it simply redirects attention and therefore cannot easily compensate for demographic underrepresentation [33]. When balancing the data set, our methods did not include undersampling due to our small data sets, however, this may be a potential avenue for future research.

Second, we considered featurization and highlighted sex differences in the biochemical manifestation of disease. In current clinical practice, the diagnostic parameters used for identifying pathology are drawn from research trials dominated by male physiology: it is perhaps unsurprising therefore that algorithms built from these data tend to underperform in female disease. There is a growing body of research that critiques the use of unisex thresholds in medicine for biochemical tests; our sex-stratified analysis of the cardiac data sets and the identified sex differences in feature rankings supports these proposals [16].

There are further sources of inequitable performance that our evaluation cannot distinguish between. It may be that the sex differences in the physiological expression of disease mean that the prediction is harder to extract from 1 population. As a result, 1 sex may require more complex models than another, with differing architecture and degrees of flexibility. It may also simply be that there are differences in the predictability of 1 group compared with another, such that if the physiology of 1 group is more opaque, it may ultimately not be possible to resolve the observed disparities. McCradden and colleagues [34] detail this challenge further in their review, highlighting that differences across groups may not always indicate inequity. There are complex causal relationships between biological, environmental, and social factors that underpin the differences in disease rates seen across population subgroups [34]. While models must not promote different standards of care according to protected characteristics, differences between groups may not necessarily reflect discriminatory practice [34].

Our research was limited by the available information in the data sets. The absence of race or ethnicity data precluded the evaluation of their effects. Furthermore, the absence of other demographic data in the studies we identified prevented the investigation of health inequities that might impact the LGBTQ+ (lesbian, gay, bisexual, transgender, queer) community, disadvantaged socioeconomic groups, or other subgroups. Previous research has described historic and institutional biases that contribute to worse health outcomes for these groups, and evolving AI systems require the same scrutiny to ensure these harms do not become embedded within digital systems [35-37].

Throughout this paper, we have used the terms male and female to reference biological sex, so as not to conflate sex and gender. With the ongoing problematic conflation of sex and gender in medicine, stratification of model performance by either sex or gender is often impossible, which was noted in our own work [35-37]. Beyond the features discussed above, there is a wide range of additional factors that we cannot account for. For example, creatinine phosphokinase was a key feature in HF modeling yet existing studies have demonstrated the variation in these levels for manual laborers and athletes, illustrating how occupation may impact a patient’s physiology [38].

To account for the complex interactions that potentiate disease, and the heterogeneous nature of patient cohorts, we require more complex modeling capable of capturing the full range of intersecting factors influencing patient health (eg, sex differences may be mediated by income). Unsupervised high-dimensional representation learning may be the path forward for this purpose [39]. In addition to improving representation, unsupervised techniques enable us to detect neglected subpopulations without predetermining a characteristic of interest, facilitating the identification of the previously overlooked disadvantaged. In this sense, AI may provide a route forward to uncovering and addressing bias, by deploying more complex modeling that can improve patient representation and by revealing previously neglected disparities in the provision of care.

### Conclusions and Limitations

In our paper, we have identified inequities in the performance of cardiac ML algorithms. Our findings are limited by the small size of the uncovered data sets, reducing their potential generalizability, and hence we propose that larger studies focused on this issue are required. These data sets also came from the same source, as we found a limited number of open-access databases due to the confidential nature of patient data and issues of proprietary ownership. In addition, we focused on RF models to replicate the papers uncovered in our literature search; however, ML models may differ in their degrees of performance disparity, and an evaluation across the range of ML model options is an important next step.

In our paper we did not attempt to solve bias; instead, we highlighted a problem that exists throughout cardiology that requires further attention. The issue we have identified in these ML models is a foundational problem across medical modeling, in any instance where the use of an “average” is applied to a diverse population. It is possible that unsupervised ML and complex representational modeling may be a route forward for capturing heterogeneity in a previously unattainable manner and addressing issues of bias [39]. Our findings demonstrate that examining performance inequities across demographic subgroups is an essential approach for identifying biases in AI and preventing the perpetuation of inequalities in digital health systems.

## Appendix

Multimedia Appendix 1 Details of literature search and data sets.

Multimedia Appendix 2 Details of Fair Adversarial Gradient Tree Boosting.

## Abbreviations

AI: artificial intelligence
CAD: coronary artery disease
DispFNR: disparity in false negative rate
EF: ejection fraction
FAGTB: Fair Adversarial Gradient Tree Boosting
FNR: false negative rate
FPR: false positive rate
HF: heart failure
LGBTQ+: lesbian, gay, bisexual, transgender, queer
ML: machine learning
PRISMA: Preferred Reporting Items for Systematic Reviews and Meta-Analyses
RF: random forest
