# Supplementary material for: Sex-Based Performance Disparities in Machine Learning Algorithms for Cardiac Disease Prediction: Exploratory Study
Source: J Med Internet Res. 2024 Aug 26;26:e46936. doi: 10.2196/46936 (PMC11384168; doi:10.2196/46936)
Supplement: Multimedia Appendix 1 [file jmir_v26i1e46936_app1.pdf]

Multimedia Appendix 1

Details of literature search and datasets

Table S1. Literature Review Details and MESH Terms for search carried out between 1st April 2022 and 22nd May 2022 *(timespan of search Timespan: 1900-01-01 to 2022-05-22, *Article type restricted to full research papers (not isolated abstracts)*

Academic Database and MESH Terms	Number of Results
PubMed<br>((((artificial intelligence[MeSH Major Topic]) OR (machine learning[MeSH Major Topic]) OR (deep learning[MeSH Major Topic]) OR (unsupervised learning[MeSH Major Topic]) OR (supervised learning[MeSH Major Topic])) AND ((heart failure [MeSH Major Topic]) or (cardiac failure[MeSH Major Topic])) AND ((predic*[MeSH Terms])))	31 Results
Web of Science<br>(((TI=(artificial intelligence) OR TI=(machine learning) OR TI=(unsupervised machine learning) OR TI=(supervised machine learning)) AND ((TI=(cardiac failure) OR TI=(heart failure)) AND (AB=(predict*)))))	96 Results

Table S2: checklist PRISMA: Preferred Reporting Items for Systematic Reviews and Meta-Analyses

Section and Topic	Item #	Checklist item	Location where item is reported
**TITLE**			
Title	1	Identify the report as a systematic review.	Methods
**ABSTRACT**			
Abstract	2	See the PRISMA 2020 for Abstracts checklist.	Abstract
**INTRODUCTION**			
Rationale	3	Describe the rationale for the review in the context of existing knowledge.	Background
Objectives	4	Provide an explicit statement of the objective(s) or question(s) the review addresses.	Background
**METHODS**			
Eligibility criteria	5	Specify the inclusion and exclusion criteria for the review and how studies were grouped for the syntheses.	Methods
Information sources	6	Specify all databases, registers, websites, organisations, reference lists and other sources searched or consulted to identify studies. Specify the date when each source was last searched or consulted.	Methods
Search strategy	7	Present the full search strategies for all databases, registers and websites, including any filters and limits used.	Supplementary Material
Selection process	8	Specify the methods used to decide whether a study met the inclusion criteria of the review, including how many reviewers screened each record and each report retrieved, whether they worked independently, and if applicable, details of automation tools used in the process.	Methods
Data collection process	9	Specify the methods used to collect data from reports, including how many reviewers collected data from each report, whether they worked independently, any processes for obtaining or confirming data from study investigators, and if applicable, details of automation tools used in the process.	Methods
Data items	10a	List and define all outcomes for which data were sought. Specify whether all results that were compatible with each outcome domain in each study were sought (e.g. for all measures, time points, analyses), and if not, the methods used to decide which results to collect.	Methods & Supplementary
	10b	List and define all other variables for which data were sought (e.g. participant and intervention characteristics, funding sources). Describe any assumptions made about any missing or unclear information.	Methods & Supplementary
Study risk of bias assessment	11	Specify the methods used to assess risk of bias in the included studies, including details of the tool(s) used, how many reviewers assessed each study and whether they worked independently, and if applicable, details of automation tools used in the process.	Methods & Supplementary
Effect measures	12	Specify for each outcome the effect measure(s) (e.g. risk ratio, mean difference) used in the synthesis or presentation of results.	N/A
Synthesis methods	13a	Describe the processes used to decide which studies were eligible for each synthesis (e.g. tabulating the study intervention characteristics and comparing against the planned groups for each synthesis (item #5)).	Methods & Supplementary
	13b	Describe any methods required to prepare the data for presentation or synthesis, such as handling of missing summary statistics, or data conversions.	Methods & Supplementary
	13c	Describe any methods used to tabulate or visually display results of individual studies and syntheses.	Methods & Supplementary
	13d	Describe any methods used to synthesize results and provide a rationale for the choice(s). If meta-analysis was performed, describe the model(s), method(s) to identify the presence and extent of statistical heterogeneity, and software package(s) used.	Methods & Supplementary
	13e	Describe any methods used to explore possible causes of heterogeneity among study results (e.g. subgroup analysis, meta-regression).	Methods & Supplementary
	13f	Describe any sensitivity analyses conducted to assess robustness of the synthesized results.	N/A

Section and Topic	Item #	Checklist item	Location where item is reported
Reporting bias assessment	14	Describe any methods used to assess risk of bias due to missing results in a synthesis (arising from reporting biases).	Methods & Supplementary
Certainty assessment	15	Describe any methods used to assess certainty (or confidence) in the body of evidence for an outcome.	Methodology & Discussion
**RESULTS**			
Study selection	16a	Describe the results of the search and selection process, from the number of records identified in the search to the number of studies included in the review, ideally using a flow diagram.	Results & Supplementary
	16b	Cite studies that might appear to meet the inclusion criteria, but which were excluded, and explain why they were excluded.	Results & Discussion
Study characteristics	17	Cite each included study and present its characteristics.	Results
Risk of bias in studies	18	Present assessments of risk of bias for each included study.	Results
Results of individual studies	19	For all outcomes, present, for each study: (a) summary statistics for each group (where appropriate) and (b) an effect estimate and its precision (e.g. confidence/credible interval), ideally using structured tables or plots.	N/A
Results of syntheses	20a	For each synthesis, briefly summarise the characteristics and risk of bias among contributing studies.	N/A
	20b	Present results of all statistical syntheses conducted. If meta-analysis was done, present for each the summary estimate and its precision (e.g. confidence/credible interval) and measures of statistical heterogeneity. If comparing groups, describe the direction of the effect.	N/A
	20c	Present results of all investigations of possible causes of heterogeneity among study results.	N/A
	20d	Present results of all sensitivity analyses conducted to assess the robustness of the synthesized results.	N/A
Reporting biases	21	Present assessments of risk of bias due to missing results (arising from reporting biases) for each synthesis assessed.	N/A
Certainty of evidence	22	Present assessments of certainty (or confidence) in the body of evidence for each outcome assessed.	N/A
**DISCUSSION**			
Discussion	23a	Provide a general interpretation of the results in the context of other evidence.	Discussion
	23b	Discuss any limitations of the evidence included in the review.	Discussion
	23c	Discuss any limitations of the review processes used.	Discussion
	23d	Discuss implications of the results for practice, policy, and future research.	Discussion
**OTHER INFORMATION**			
Registration and protocol	24a	Provide registration information for the review, including register name and registration number, or state that the review was not registered.	Methods
	24b	Indicate where the review protocol can be accessed, or state that a protocol was not prepared.	N/A
	24c	Describe and explain any amendments to information provided at registration or in the protocol.	N/A
Support	25	Describe sources of financial or non-financial support for the review, and the role of the funders or sponsors in the review.	Methods
Competing interests	26	Declare any competing interests of review authors.	Title Page
Availability of data, code and other	27	Report which of the following are publicly available and where they can be found: template data collection forms; data extracted from included studies; data used for all analyses; analytic code; any other materials used in the review.	Methods

Section and Topic	Item #	Checklist item	Location where item is reported
materials			

Table S3: description of features for data set 1 (heart failure)

The 'Heart Failure (HF) Clinical Records Dataset' was imported from the UCI institute; the available clinical/biochemical features and target variable (patient death) are detailed in below. The dataset contains records of 299 HF patients collected at the Allied Hospital in Faisalabad (Punjab, Pakistan) during 2015. UCI; University of California Irvine.

	**Description**	**Measurement**	**Datatype**
**Age**	Age of the patient	Years	Interger, continuous
**Anaemia**	Reduced count of red blood cells or haemoglobin	Yes/No	Integer, binary
**High blood pressure**	Whether the patient has high blood pressure	Yes/No	Integer, binary
**Creatinine phosphokinase (CPK)**	The level of CPK enzyme in the blood	Micrograms/litre (mcg/l)	Float(64), continuous
**Diabetes**	Previous diagnosis of diabetes	Boolean (0, 1)	
**Ejection Fraction**	The percentage of blood that is ejected from the heart with each contraction	Percentage	Numerical, continuous
**Sex**	Sex of patient	Female (0), Male (1)	Numerical
**Platelets**	Count of platelets in the blood	Kiloplatelets/ml	Numerical, continuous
**Serum creatinine**	The level of creatinine in the blood	Mg/d	Numerical continuous
**Serum sodium**	The level of sodium in the blood	mEq/L	Numerical, continuous
**Smoking**	Whether the patient smokes	Boolean<br>False (0), True (1)	Integer, binary
**Time**	Follow up period of patient	Days	Integer, continuous
**Death event (outcome variable)**	If a patient died during follow-up period	Boolean (Death (1), Survival (0)).	Integer, binary

Table S4: description of features for data set 2 (CAD data set)

The Cleveland Heart dataset is a subset of a wider database, in which we found the subset to contain only twenty unwell women. We therefore use the original full database which integrates hospital data across multiple sites Cleveland, Hungary, Switzerland and Long Beach, published by Siddartha et al and used more widely in the research literature. Full details available at: *Manu Siddhartha. (2020). Heart Disease Dataset (Comprehensive). IEEE Dataport.* *https://dx.doi.org/10.21227/dz4t-cm36*

CAD; Coronary artery disease, IEEE; (institute of electrical and electronics engineers).

	**Description**	**Measurement and Range**	**Datatype**
**Age**	Patient's age	Years (28 – 77 years)	Numerical continuous
**Sex**	Sex of patient	Female (0), Male (1)	Numerical
**Chest pain type**	Category of chest pain	Typical (1), Typical Angina (2), Non-anginal pain (3), Asymptomatic (4)	Numerical
**Resting BP S**	Level of blood pressure at rest	Mm/hg	Numerical
**Cholesterol**	Serum cholesterol	Mg/dl	Numerical
**Fasting blood sugar**	Blood sugar levels on fasting of >120mg represented as 1 if true, 0 if false	0, 1	Boolean
**Resting ECG**	Result of electrocardiogram while at rest	Normal (0), ST-T Wave abnormality (1), Evidence of Left Ventricular Hypertrophy (2)	Numerical
**Max Heart Rate**	Maximum heart rate achieved	60 – 202 Seconds	Numerical, continuous
**Exercise Angina**	Whether angina is induced by exercise	No (0), Yes (1)	Nominal
**Old Peak**	Whether there is exercise induced ST depression on the ECG in comparison with state of rest	Mm (-2.6 – 6.2)	Numerical, continuous
**ST Slope**	The slope of the peak exercise ST Segment	Upsloping (1), Flat (2), Downsloping (3)	Nominal

Table S5: Descriptive Statistics of Features Stratified by Target (CAD Diagnosis) and Sex for data set 2

The table below provides the mean and standard deviation for each of the features in data set 2 (CAD dataset). Of note, the original dataset reports a total participant count of 1190, however this fell after duplicate and null values were removed, given the total count of n=746 presented below. As described in the original file and in the table caption below, the outcome variable of CAD diagnosis assigns a value 1-5 of disease severity based on the narrowing of vessels, this is changed to a binary outcome where values above 2 are considered diseased (value 1). The dataset contains 76 attributes, but all published experiments refer to a subset of just 14 of them. CAD; Coronary artery disease.

Descriptive statistics of the variables in Dataset 2 (Coronary Artery Disease) (n=746), stratified by Target (CAD Diagnosis) and Sex

**Sex**	**Female (Sex = 0)** (n=182)				**Male (Sex = 1)** (n=564)			
**CAD Diagnosis (Target Variable)**	**Healthy (CAD Diagnosis = 0)**		**Diseased (CAD Diagnosis = 1)**		**Healthy (CAD Diagnosis = 0)**		**Diseased (CAD Diagnosis = 1)**	
Total Count (N) & Event Rate (%)	142 (78.0%)		40 (22.0%)		248 (44.0%)		316 (56.0%)	
	Mean	Std. Dev	Mean	Std. Dev	Mean	Std. Dev	Mean	Std. Dev
Age (years)	51.1	9.6	56.0	7.2	49.6	9.1	55.8	9.0
Chest pain type (1-4, Categorical)	2.7	0.9	3.7	0.7	2.8	0.9	3.6	0.8
Resting BP (Mm/hg)	128.8	16.7	143.4	20.7	131.0	15.8	135.2	17.4
Cholesterol (mg/dl)	249.2	62.2	279.2	60.1	232.8	50.2	247.5	61.9
Fasting blood sugar (boolean)	0.1	0.3	0.2	0.4	0.1	0.3	0.2	0.4
Resting ECG (0-2 Categorical)	0.6	0.8	0.8	0.9	0.5	0.8	0.7	0.8
Max Heart Rate (seconds)	149.2	21.6	139.2	21.7	149.0	24.0	129.4	22.2
Exercise Angina (boolean)	0.1	0.3	0.6	0.5	0.1	0.3	0.7	0.5
Old peak (mm)	0.4	0.6	1.5	1.4	0.4	0.7	1.5	1.1
ST slope (1-2, Categorical)	1.3	0.5	2.0	0.4	1.2	0.5	2.0	0.5

**CAD = Target variable, diagnosis of Coronary Artery Disease, full details of the variables and their datatypes are provided in Supplementary Table 3. Further documentation and full Dataset available at: Manu Siddhartha. (2020). Heart Disease Dataset (Comprehensive). IEEE Dataport. https://dx.doi.org/10.21227/dz4t-cm365*

Table S6 – Training data: case counts for data set 1 (heart failure)

A summary of the different training data available in each of the training datasets that were formed.

Sex	Target (HF Death)	Original unbalanced dataset (included to represent original studies)	Balanced Dataset	Female Specific training data	Male Specific Training Data
0	0	71	153	153	X
	1	34	41	41	X
1	0	132	132	X	132
	1	62	62	X	62
	**Total**	299	388	194	194
	**Total Training (0.7*total)**	209	272	136	136

**Unlike below, no erroneous or duplicate records needed to be removed*

Table S7 – Training Data: Case counts for data set 2 (Coronary Artery Disease)

A summary of the different training data available in each of the training datasets that were formed.

Sex	Target (CAD Diagnosis)	Original Cleveland Dataset	Dataset 2 (Combined dataset of five hospitals)	Dataset 2, (Duplicates values removed)	Final Unbalanced Training Dataset (erroneous values removed*)	Final Balanced Training Dataset (including female and male training subsets)
0	0	72	211	143	142	248
	1	25	70	50	40	263
1	0	92	350	267	248	248
	1	114	559	458	316	263
**Total Data**		303	1190	918	746	1022
**Total Training Data (n*0.7)**		212	833	643	522	715

**Erroneous values included 172 instances Cholesterol = 0, and 1 instance Resting blood pressure = 0.*

**For sex specific training samples the training data was the sex subset of the balanced training data, i.e. for females 248 well and 263 unhealthy. Hence, total females/males in Final Balanced Training Data = 511, Females/males in respective sex-specific subset (511*0.7 = 358).*

Table S8: Feature Subsets for dat aset 1 (Heart Failure)

Features with Sex	Features without Sex	Clinical Features Only	Biochemical Features Only
Sex	Age	Anaemia	CPK
Age	Smoking	Diabetes	Serum Creatinine
Anaemia	Anaemia	Ejection Fraction	Platelets
CPK	CPK	High Blood Pressure	Serum Sodium
Diabetes	Diabetes		
Ejection Fraction	Ejection Fraction		
High blood pressure	High blood pressure		
Platelets	Platelets		
Serum Creatinine	Serum Creatinine		
Serum sodium	Serum sodium		
Smoking			

Table S9: Feature Subsets defined for data set 2 (Coronary Artery Disease)

Features with Sex	Features without Sex	Clinical Features Only	Biochemical Features Only
Cholesterol	Cholesterol	Chest pain type	Cholesterol
Fasting blood sugar	Fasting blood sugar	Resting BP	Fasting blood sugar
Age	Age	Resting ECG	
Chest pain type	Chest pain type	Max Heart Rate	
Resting BP	Resting BP	Exercise Angina	
Resting ECG	Resting ECG	Old peak	
Max Heart Rate	Max Heart Rate	ST Slope	
Exercise Angina	Exercise Angina		
Old peak	Old peak		
ST Slope	ST Slope		
Sex			
