# Supplementary material for: Sex-Based Performance Disparities in Machine Learning Algorithms for Cardiac Disease Prediction: Exploratory Study
Source: J Med Internet Res. 2024 Aug 26;26:e46936. doi: 10.2196/46936 (PMC11384168; doi:10.2196/46936)
Supplement: Multimedia Appendix 2 [file jmir_v26i1e46936_app2.pdf]

Multimedia Appendix 2: Details of Fair Adversarial Gradient Tree Boosting.

Section S1: Details of FAGTB. FAGTB: Fair Adversarial Gradient Tree Boosting.

Summary of Fairness Metrics

The authors propose using a fairness regulariser that aims to remove correlation between the sensitive attribute and the target value [7]. The objective of the algorithm is to predict the target variable Y with gradient tree boosting, while minimizing the ability of an adversarial neural network to predict the sensitive attribute (sex).

The authors focus on two definitions of fairness Demographic Parity and Equalized Odds. The Equalized Odds metric focuses on model false positive and false negative error rates. For Equalised Odds the algorithm is considered fair if across both demographics, for outcome $\hat{Y} = 1$ the predictor $\hat{Y}$ has equal true positive rates, and for $\hat{Y} = 0$ the predictor $\hat{Y}$ has equal false positive rates. As a result, this metric frames fairness as the chance of being correctly or incorrectly classified, as a positive should be equal for every group.

1) Demographic Parity

To achieve fairness the authors focus on *Demographic Parity,* for which a classifier is considered fair if the prediction $\hat{Y}$ from features X is independent from the protected attribute S. The foundational idea is that each demographic group has the same change of a positive outcome (Equation 1).

Equation 1, Definition of Demographic Parity: $P(\hat{Y} = 1 \,|\, S = 0) = P(\hat{Y} = 1 \,|\, S = 1)$

The authors define multiple ways to assess this objective, we focus on the p-rule, DispFNR and DispFPR.

P-Rule

The p-rule assessment ensures the ratio of the positive rate for the unprivileged group is no less than a fix threshold $\frac{p}{100}$. A classifier is considered completely fair when this ratio satisfies a 100% rule, whereas a 0% rule indicates a completely unfair model.

Equation 2, P Rule: $\min\left(\frac{P(\hat{Y}=1 \,|\, S=1)}{P(\hat{Y}=1 \,|\, S=0)}, \frac{P(\hat{Y}=1 \,|\, S=1)}{P(\hat{Y}=1 \,|\, S=0)}\right)$

Disparate Impact

The authors also use disparate impact (DI) assessment as a second metric for evaluating demographic parity. The DI considers the absolute difference of outcome distributions for subpopulations with different sensitive attribute values, the smaller the difference, the fairer the model.

Equation 3, DI: $|P(\hat{Y} = 1 \,|\, S = 1) - P(\hat{Y} = 1 \,|\, S = 0)|$

2) Equalised Odds

In the context of Equalised Odds, the algorithm is considered fair if across both demographics, for outcome $\hat{Y} = 1$ the predictor $\hat{Y}$ has equal true positive rates, and for $\hat{Y} = 0$ the predictor $\hat{Y}$has equal false positive rates. As a result, this metric frames fairness as the chance of being

correctly or incorrectly classified, as a positive should be equal for every group. As presented in Equation 4, the Equalised Odds states that the true positives for one demographic group (S=0), should equal the true positives for the other demographic group (S=1).

Equation 4, Definition of Equalised Odds: $P(\hat{Y} = 1 \,|\, S = 0, Y = y) = P(\hat{Y} = 1 \,|\, S = 1, Y = y), \forall y \in \{0,1\}$

To assess for the Equalised Odds the authors measure the Disparate Mistreatment (DM), which computes the absolute difference between the false positive rate (FPR) and the false negative rate (FNR) for both demographics.

Equation 5, Disparate False Positive Rate:

$$D_{FPR} : |\, P(\hat{Y} = 1 \,|\, Y = 0, S = 1) - P(\hat{Y} = 1 \,|\, Y = 0, S = 0)|$$

Equation 6, Disparate False Negative Rate:

$$D_{FNR} : |\, P(\hat{Y} = 0 \,|\, Y = 1, S = 1) - P(\hat{Y} = 0 \,|\, Y = 1, S = 0)|$$

We replicate the bias mitigation techniques reported by Grari et al which are based on Gradient Boosted Decision Trees. As per the original paper, we repeat 10 experiments by randomly sampling two subsets (80% training and 20% test set) and report the accuracy and fairness metrics for the test set. The Fairness metrics include 'Disparate Mistreatment', Disparity FNR and Disparate FNR. In keeping with the rest of our paper, we focus on the Disparate FNR. The closer the values of DFPR and DFNR to 0, the lower the degree of disparate mistreatment of the classifier.
